# Design Improvements for Personal Protective Equipment Used in Ebola and Other Epidemic Outbreaks

**DOI:** 10.9745/GHSP-D-17-00152

**Published:** 2017-06-27

**Authors:** Margaret Glancey, Patience Osei, William Alexander Patterson, Matthew Petney, Laura Scavo, Chandrakant Ruparelia, Soumyadipta Acharya, Youseph Yazdi

**Affiliations:** a Department of Biomedical Engineering, Johns Hopkins University, Baltimore, MD, USA.; b Jhpiego, Baltimore, MD, USA.

## Abstract

We redesigned the personal protective equipment ensemble widely used during the 2014 Ebola outbreak into a relatively simpler and more versatile coverall and hood, to improve protection and usability for frontline workers treating patients in infectious disease outbreaks around the world.

## BACKGROUND

In 2014, the world witnessed the worst Ebola outbreak on record, with widespread disease in the West African countries of Guinea, Liberia, and Sierra Leone—infecting more than 28,000 people and killing more than 11,300 in the span of 2 years.^1^ It was the first Ebola outbreak to reach large city centers, where it spread rapidly. The disease particularly impacted health care workers because it is so highly infectious and difficult to defend against, placing additional pressure on already fragile health care systems. By August 2015, 880 health care workers had become infected, and 512—nearly 60%—of those workers had died.^2^

One of the greatest times of risk for health care workers occurs when they remove their personal protective equipment (PPE) and are exposed to the virus on the outer surfaces, especially during glove and gown removal (doffing). Compounding the danger is the complexity of the doffing protocols and of the multipiece PPE ensembles themselves.^3^ Every additional element and step in a multicomponent PPE ensemble, either in donning or doffing, adds an opportunity for error and increase in risk.

In October 2014, in response to these issues and to the United States Agency for International Development's (USAID's) Grand Challenge for Development to help health care workers respond to the epidemic, Johns Hopkins University's Center for Bioengineering Innovation and Design (CBID) and Jhpiego, an NGO affiliate of Johns Hopkins University, organized the “Johns Hopkins Emergency Ebola Design Challenge,” a hackathon-type event in which participants worked in teams to address specific issues with existing PPE ensembles. The event was also supported by the State of Maryland and Clinvue, a Maryland-based medical device innovation and design company. Experts from many disciplines, including experts in the care of Ebola patients and use of PPE, were brought together for the event, which was aimed at understanding and designing mitigations to a wide range of issues pertaining to use of PPE in infectious disease outbreaks like Ebola. More than 80 people participated throughout the 3-day event, and teams were formed for continued development for many weeks after. They addressed such issues as doffing, donning, comfort, visibility, fogging, patient/family fear of caregivers, and overall complexity of protection from the virus. More than 100 concepts to address these issues were generated. After additional development and refinement, the leading concepts were combined into a set of new PPE designs. Further development was supported by a grant from USAID, a partnership with DuPont Corporation, and additional support from CBID and Jhpiego. Here, we discuss the new improved features of the PPE (hood and coverall) and current commercialization prospects.

## THE DESIGN

Traditional PPE used by Médecins Sans Frontières (MSF) consists of a coverall or gown to protect the body, a separate hood to cover the head, a mask to cover the mouth and nose while allowing for breathing (typically a duckbill N95 respirator and surgical mask), laboratory-type goggles to cover the eyes, 2 layers of gloves for the hands and forearms, rubber boots, and often a heavy plastic apron in front. Ideally, this ensemble is worn in a way that completely covers the skin of the health care worker: the goggles overlap with the face mask, the hood has to overlap the coveralls, etc. Any gap could expose the health care worker to infectious agents.

Multiple studies have described the challenges associated with PPE, especially during doffing, when the risk of contamination is highest. These challenges include contamination of hands and wrists during glove removal, exposure and contamination of the neck during doffing, overheating and discomfort, gowns opening while in use, and a restricted field of vision, made worse by fogging inside the goggles.^4^^,^^5^ Additional elements of complexity include the many steps in the protocol to don and doff this ensemble, as well as the multiplicity of sources for the products in the ensemble.

Health care workers face many challenges with personal protective equipment that must be worn during epidemic outbreaks, especially during doffing when the risk of contamination is highest.

In the later stages of the response to an outbreak, the risks associated with these issues are minimized, as training and experience reach high levels. In the earliest stages of an outbreak, however, when health care workers are just learning how to use PPE, such complexity and design weaknesses may add significantly to the magnitude of the risk, and subsequently, to the magnitude and trajectory of the crisis.

CBID teams obtained feedback throughout the design process from more than 200 health care workers (hygienists, medics, health experts, midwives, maternal and obstetrics and gynecology experts, PPE trainers) at the Johns Hopkins Bio Containment Unit, Jhpiego, and health facilities in West Africa, including clinicians who had worked in Ebola Treatment Units. Additional insights were gathered through visits to MSF headquarters in Brussels and to the World Health Organization (WHO) in Geneva.

**FIGURE 1:**
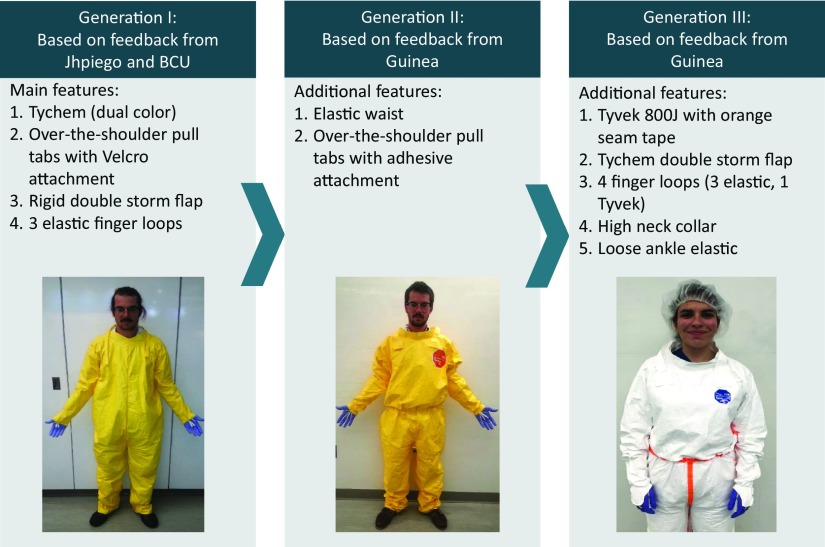
Design Iterations to Personal Protective Equipment Coveralls Abbreviation: BCU, Johns Hopkins Bio Containment Unit.

**FIGURE 2:**
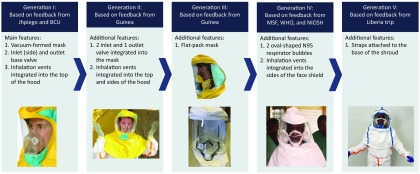
Design Iterations to Personal Protective Equipment Hood and Respirator Abbreviations: BCU, Johns Hopkins Bio Containment Unit; MSF, Médecins Sans Frontières; NIOSH, National Institute for Occupational Safety and Health; WHO, World Health Organization.

With an understanding and deeper assessment and ranking of the various problems facing health care workers, our team focused further development efforts on concepts that simplify the doffing procedure, reduce the risk of contamination during use, improve comfort, improve visibility of the patient to the caregiver's face, improve the vision of the wearer, and reduce the number of steps and parts. Multiple iterations of the improved coverall and hood were produced based on this feedback, and design refinements were regularly shared with DuPont, the product commercialization partner (PCP). DuPont partners further refined the PPE design, incorporating factors such as cost and manufacturability, and then built the next set of prototypes (Figure 1 and Figure 2).

We assembled a design team to improve the personal protective equipment worn by health care workers during outbreaks.

### Final Design

The final ensemble was reduced to 3 product concepts:

1. An improved coverall, covering the user from the neck down
2. An improved hood, which would replace several parts and functions of the existing hood/goggle/mask ensemble covering the head and face
3. A combined single product incorporating the coverall and hood

The new coverall aimed to improve ease of doffing and reduce the risk of contamination during the process. Elements of the coverall design included enabling a cocoon-type doffing technique, a rear zipper, doffing tabs, protective flaps for the zipper, simplified labeling with instructions, finger loops to reduce risk of wrist exposure, and others. In the new hood design, elements included a clear, large face shield to improve visibility and comfort of use, reengineered air flow pattern that allowed fresh air to flow in over the inside of the face shield to reduce fogging, straps attached to the base of the shroud to secure it to the waist and aid during doffing, and a simpler combination that is more intuitive to use and has fewer separate parts, to simplify protection especially in the earliest stages of an outbreak. A training video demonstrates the features and donning and doffing of the new PPE: https://www.youtube.com/watch?v=App79IR0Y-c.

The redesigned coverall, with a rear zipper, doffing tabs, finger loops to reduce risk of wrist exposure, and several other improved elements, aimed to improve ease of doffing and reduce the risk of contamination during the process.

Doffing safety and time was improved by reducing the number of steps and simplifying the process dramatically. A rear-entry dual zipper protected by a double storm flap to prevent contamination and accidental unzipping was added to the back of the suit. The double storm flaps were reinforced with a rigid material to maintain configuration during use. Other features include doffing pull-tabs at the crown of the head, elastic seams at the waist, and fingerless gloves that keep inner gloves in place during removal of the coverall and outer gloves. Preliminary unpublished results from a small usability study showed that more than 75% of participants at field sites in Liberia and the United States found the doffing process for the new coverall and hood easier and more intuitive than a current standard PPE suit and doffing protocol.

Doffing safety and time was improved with the redesigned personal protective equipment by reducing the number of steps and simplifying the process.

## MARKETING AND COMMERCIALIZATION

A key objective of the project was identifying a PCP early on in the project. Typically, for academic-based innovation and design efforts, considerable amount of work is done prior to engaging an outside PCP. Part of the CBID Innovation Model is to engage the PCP and other key stakeholders from the very beginning of the project to ensure that what is designed is something the PCP is eager to commercialize. Given the considerable investment and employee time required to bring a new product to market, such early and sustained interaction significantly increases the likelihood of commercialization. In this project, after considered requests from several PPE manufacturers, DuPont was engaged as a partner under an agreement that the company would introduce products based on these designs into its normal product development and commercialization process, contribute design elements to reduce costs and improve performance, and prepare prototypes for use during the design-feedback iterations.

An important design target during the development process was to produce a design that could be manufactured and sold in large volumes at a price comparable to that of current PPE ensembles. The coverall and hood will use similar materials and manufacturing as is currently in use, and best estimates are that such price is not to be more than 20% higher than the price of existing designs. Manufacturing of the third product concept, the full body suit, will follow the final design and manufacturing of the coverall and hood.

## CHALLENGES AND NEXT STEPS

At the conclusion of the USAID-funded part of the project, CBID design teams transferred designs, data, and future plans to DuPont and USAID. DuPont remains committed to manufacture and make available to their customers products based on concepts developed by this project. Prototypes of the coverall and hood products have undergone Department of Defense (DoD) testing and preliminary Centers for Disease Control and Prevention (CDC) testing (results have not been published yet). Preliminary results showed some improved performance as well as areas that could benefit from continued refinement.

Several challenges have arisen during the project. One involves changes pertaining to the regulatory clearance process for PPE, for both the designs and materials, in the view of the US Food and Drug Administration (FDA). Note that new material development was not the focus of this project. Additionally, other organizations that provide standards and guidance for PPE, primarily WHO, have been working to modify and refine those standards (including material selection, donning, doffing, and future product needs) over the years since the first outbreak. The identification and publication of these standards, and build-up of demand for better PPE products, will help accelerate company investment in production. It is anticipated that the coverall will enter large-scale manufacturing by the end of 2017. The new hood will continue to undergo technical development by DuPont based on results of usability studies.

In conclusion, using the tools and methodology of biomedical engineering and product design, including short-time Design Challenges as well as sustained work by Design Teams, and engaging all key stakeholders early on in an iterative design process, this team was able to design a new safer, simpler, and versatile PPE coverall and hood that has the potential to improve protection of frontline health care workers treating patients in infectious disease outbreaks around the world.

## Notes

**Peer Reviewed**
